# Modeling and forecasting the volatility of some industry development indicators in Ethiopia using multivariate GARCH models

**DOI:** 10.1038/s41598-024-64749-3

**Published:** 2024-06-22

**Authors:** Getachew Abate Dagnew, Birhan Walie Alamneh, Wosenie Gebireamanuel Hailu

**Affiliations:** 1 Department of Statistics, Mekdela Amba University, Tulu Awulia, Ethiopia; 2 Department of Statistics, Bahir Dar University, Bahir Dar, Ethiopia; 3 Department of Statistics, Wollo University, Dessie, Ethiopia

**Keywords:** Environmental social sciences, Mathematics and computing

## Abstract

Industry development indicators refer to a set of measures used to assess the performance and growth of industries. The main aim of this study was to assess the relationship between industry development indicators in Ethiopia using a multivariate GARCH model based on World Bank data from 1982 to 2021. A time series technique using annual data for the period 1982–2021 is utilized, and multivariate generalized autoregressive conditional heteroscedasticity was performed for volatility modeling. The results of the diagonal BEKK (1, 1)-GARCH model showed that there is strong evidence for a GARCH effect and the presence of a weaker ARCH effect, Equations show a statistically significant co-variation in shocks, which depends more on their lags than on past errors. Consequently, development indicator shocks are influenced by past information. The cross-volatility effects are higher than the own-volatility effects in Industry GDP, manufacturing GDP, and manufacturing exports. However, past volatility shocks in industry growth have less effect on cross-volatility than its own volatility shock. The implication of the study is that both domestic policymakers and development partners should support and motivate the growth of manufacturing sectors and manufacturing exports, since this is a necessary condition for promoting industry growth.

## Introduction

Industrialization, as history has shown, is essential for economic growth. A structural shift from conventional to modern industrial sectors boosts output, encourages creativity, speeds up the spread of new technologies, and has other advantageous knock-on effects^1^. Any economic activity that includes the production of goods, the acquisition of service, and the provision of services is referred to as an industry^2^.

Industrialization has had a substantial impact on economic growth in countries like China, the Republic of Korea (Korea), the Taiwan Province of China (Taiwan), and Indonesia. Along with rapid growth, poverty rates have declined in many countries. Inequality has worsened in certain countries while remaining an issue in others^3^.

Ethiopia is one of the few African countries that has formulated and implemented a full-fledged industrial development strategy since the early 2000s, when industrial policy was taboo in international policy forums. The industry's manufacturing sector must expand in order to build national technological capacity, manufacturing capability, technological progress, productivity, and capital accumulation^4^.

Manufacturing is described as the physical or chemical transformation of material components into new products. The definition also includes the assembly of various elements of manufacture Products as a manufacturing activity, whether the production is done at a factory or at home, sold at retail or wholesale, and whether a power-driven machine is used or not^5^. Currently, Sub-Saharan Africa has the lowest per-capita manufacturing output of any inhabited region on the planet. Most African economies, in contrast to the newly industrialized (NIC) Asian countries, have so far failed to supplement agricultural and extractive output by raising average productivity through the creation of a substantial number of jobs in higher-value-added manufacturing sectors^6^.

Ethiopian manufacturing sectors contribute a great deal to exports, employment, and national output. Currently, Ethiopia is ranked among the five fastest-growing economies in the world. However, the sectors within manufacturing in Ethiopia are still in their infancy due to the shortage of skilled workers, lower competitiveness, lack of advanced technology, and sustainability awareness^7^.

Manufacturing firms are viewed as an essential element of a healthy and vibrant economy. They are seen as vital to the promotion of an enterprise culture and to the creation of jobs within the financial system^8^. Industry manufacturers are believed to provide an impetus to the economic progress of developing nations, and their importance is gaining widespread recognition^9^. There are several industrial development indicators, like industrial output, value added, employment, wages, industrial labor force, and manufactured and semi- manufactured exports at the aggregated sector level; GNP, population, geographical area, and total merchandise trade; and human resources: skills, education, and nutrition^10–12^.

The industry promotes various aspects of social integration through its general thrust towards modernization and makes a specific contribution to national economic integration^13^. From this, four features can be deduced. First, industrialization is not a one-time occurrence but rather a sustained process. Second, industrialization requires the application of modern science and technology to the production process. Third, the manufacturing sector plays the most important role in the industrialization process; that is, the key and dynamic roles in this process are played by the manufacturing sector. Last but not least, industrialization brings about a structural transformation of the national economy. As a result, this makes the manufacturing sector a dynamic component of the industrial sector^14^.

Growth in industrial manufacturing can be possible only through external demand with a high growth rate, that is, through export. The higher the growth rate in the manufacturing industry that exports determine, the faster the transfer of labor will be from sectors in which economic productivity is low to the industrial sector, which leads to a faster productivity increase^15^. Therefore, studying the linkage between manufacturing exports and various development indicators helps us understand the status of industry growth and the causes of manufacturing growth in Ethiopia.

Thus, the main aim of this study was to investigate the volatility and relationship between industry development indicators in Ethiopia from 1982 to 2021. Specifically, this research attempts to address the trends in industry development indicators, fit volatility models, and determines the appropriate one for the data and to forecast the volatility of industry development indicators for the next five years.

Ethiopia’s manufacturing sector was growing in terms of the number of firms, employment, output, value addition, exports, and investment. Growth figures appear to be high, mainly because of the country’s low industrial base. In practice, the sector has not grown as per the country’s growth potential and goals as stipulated in Growth and Transformation Plan-I (GTP-I) and GTP-II. For instance, its share in GDP remained low (only 7.4% in 2019, well below the African average and far below the 17% envisioned in GTP II). Exports grow slowly and erratically, with still around 10% of the export proceeds for the country^16^. Ethiopia is virtually at the beginning of the manufacturing industry's expansion^17^. Therefore, the manufacturing sector has an impact on both industry and overall economic growth. Thus, it is better to place too much emphasis on the impact of manufacturing exports on the annual growth of the industry, and all the above facts were the motive for investigate this issue.

As far as our knowledge is concerned, some studies related to manufacturing industry growth and the relationship between development indicators have been done in Ethiopia using fixed effect regression model, tobit regression model, multiple regression model and co-integration analyses^7,14,18–22^. In addition, there are many studies done in Indonesia, Sub-Saharan Africa, East Java Province, Europe, Nigeria, and Pakistan using panel data regression with the OLS model, generalized autoregressive conditional heteroscedasticity model, fixed effect regression model, co-integration tests, and a bounds testing approach to co-integration^6,10,13,23–27^. But most of them emphasize manufacturing growth and its determinants rather than examining its instability, and they do not see the relationships and trends of many indicators simultaneously. In addition, the aforementioned studies showed that manufacturing growth itself and the effects of manufacturing growth on industrial development are subject to regime shifts^23^.

Thus, this study addressed the volatilities and the relationships of industry development indicators using the Multivariate Generalized Autoregressive Conditional Heteroscedasticity (MGARCH) models.

## Hypothesis tests

### Hypothesis 1:

The industry development indicators are stationary.

### Hypothesis 2:

The multivariate GARCH model will be able to capture the volatility of the industry development indicators.

### Hypothesis 3:

The tests for significance will be statistically significant.

### Literature review

#### Theoretical literature review

Industry is an economic activity that deals with the processing of raw materials, the manufacture of goods in factories, and the services that go along with their use. In its most restricted sense, the term "industry" may only refer to manufacturing- the making of goods, but in its broadest sense, it can refer to all phases and forms of economic activity, such as resource extraction, construction, and providing services^28^.

In order to come to a consensus on the link between manufacturing and industry growth, the industrial revolution brought about four significant transformations. Economic systems based on large-scale industry, automated manufacturing, and the factory system took the place of economies based on agriculture and handicrafts throughout the Industrial Revolution. Existing industries become more productive and efficient as a result of new equipment, power sources, and organizational techniques. The four industrial eras are coal, gas, electronics and nuclear, and the internet and renewable energy^29^. These revolutions were summarized in the following ways:

The first industrial revolution started in 1760 with the invention of the steam engine. The invention of the steam engine made it possible to go from farming and a feudal society to a modern manufacturing system. This transition included the use of coal as the main energy while trains were the main means of transportation. The two industries that dominated in terms of employment, output value, and capital invested were textile and steel^30^.

Since there is no clear distinction between the two revolutions from a technological and sociological standpoint, the second industrial revolution is sometimes known as the second phase of the industrial revolution. It was, in many ways, the continuation of the first. In many industries there was direct continuity. Yet it differed from it in a number of crucial aspects. First, it had a direct effect on real wages and standards of living which clearly differed significantly in 1914 from 1870. Second, it maintained the monopoly of the industrialised Western world on technological leadership while moving the geographic focus away from Britain to a more dispersed locus. Finally, it permanently altered how technological change occurs by altering the relationship between knowledge of nature and how it affected technological practices^31^.

The Third Industrial Revolution started in the 1950s, peaked in the dot.com period in the late 1990s, and is still continues at present. IR3 is anticipated to come to an end in the 2030s. The IR3 is seen as the transition from mechanical and analog electronics to digital electronics, which includes dispersed manufacturing, green buildings, and electric vehicles. It is based on the internet, digital technology, and the energy shift. The IR3 emerges from the corporate industry’s opportunity brought by nanotechnology, intelligent systems, 3D printing and robotics for industrial production and domestic services^32^. The third industrial revolution, often referred to as the digital revolution, which started in the late 1900s, is distinguished by the development of automation and digitalization through the use of electronics and computers, the invention of the internet and the discovery of nuclear energy. This era witnessed the rise of electronics like never before, from computers new technologies that allow the automation of industrial operations. Telecommunications development paved the groundwork for extensive globalization, which in turn enabled industries to offshore production to low-cost economies and radicalize business models worldwide^32^.

The design of computer-generated products and three-dimensional (3D) printing, which can produce solid objects by stacking successive layers of materials, are now two aspects of the fourth industrial revolution^33^. It is characterized by a fusion of technologies that is blurring the lines between the physical, digital, and biological spheres. There are three reasons why today’s transformations don't just reflect a continuation of the Third Industrial Revolution but rather the arrival of a Fourth and distinct one: velocity, scope, and systems impact. There is no historical parallel for the rapidity of modern advances. Comparing the Fourth Industrial Revolution to prior ones, it is growing exponentially rather than linearly. Almost every industry worldwide is also being disrupted by it. Additionally, the magnitude and scope of these changes denote a complete transformation of the production, management, and governance systems^34^.

Prisecaru^35^ shows a short presentation of the industrial revolutions from 1760 to the present. The main characteristics of industrial revolution-I from the period 1760–1900 and the transition period 1860–1900, the energy resource was coal, main technical achievement was steam engine internal, main developed industries were textile, steel and metallurgy and the accessed transport was train, industrial revolution-II from the period 1900–1960 and transition period 1940–1960 were characterized by oil electricity energy source, combustion engine main technical achievement, auto, machine building main developed industries and train and car transport means were utilized.

From the period 1960–2000, industrial revolutions were introduced, and the transaction period between 1980–2000, which is characterized by nuclear energy and natural gas used as energy sources, computers, robots, internets, and 3D were the main technical achievements, and auto and chemistry were the main development industries with car and plane transport means. The fourth industrial revolution from the period 2000–present, characterized by green energy resources, printers, and genetic engineering as technical achievements, developed high-tech industries with electric cars, and ultra-fast trains were used as transport mechanisms^35^.

There are also theories that justified by drawing upon several key theoretical frameworks and concepts. Here are some theoretical justifications that can support the research objective outlined in the title:

##### Financial economics theory

Financial Economics Theory is a branch of economics that focuses on understanding how financial markets, institutions, and instruments operate and how they impact economic outcomes? It applies economic principles and theories to analyze financial decisions, asset pricing, risk management, and the allocation of resources in financial markets. One of the foundational theories in financial economics is the Efficient Market Hypothesis (EMH), which posits that financial markets are efficient in reflecting all available information, making it impossible to consistently outperform the market through active trading or investment strategies. This theory has important implications for understanding asset pricing and the behavior of financial markets^36^.

##### Risk management theory

Risk management theory is a field of study that focuses on understanding and managing risks in various contexts, including finance, business, industry, and economics. It involves identifying, assessing, and mitigating risks to achieve organizational objectives and protect against potential losses. One of the foundational theories in risk management is Value at Risk (VaR), which quantifies the maximum potential loss that a portfolio or investment may face over a specified time horizon at a given confidence level. VaR is widely used in financial risk management to measure and monitor market risk^37^.

##### Forecasting theory

Forecasting theory is a set of principles and methods used to make predictions about future events or trends based on historical data and other relevant information. It involves analyzing patterns and relationships in data to make informed projections about what may happen in the future^38^.

#### Empirical literature review

According to^10^ studies using time series analysis and dynamics indicators, the world leader in terms of industrial production is China, accounting for 28.4% of world industrial production. The second and third places are occupied by the USA and India. The fourth place is occupied by Russia, so in 2021 industrial production increased by 2.3%. The highest values of the studied indicator can be traced in the following countries: Belarus, Germany, and Japan. This suggests that the manufacturing sector makes a significant contribution to the development of the country's gross domestic product. If the studied indicators have a positive development tendency and the industrial output itself appears to be promising, then the current development trend contributes to economic growth and the independence of the state.

Zhong^12^ studies on linear analysis of the correlation between industrial development and foreign trade development in Guangdong Province show that the degree of synergy between the developments level of the secondary industry in Guangdong Province and the indicators of foreign trade is relatively high, indicating that foreign trade can significantly promote the development level of the secondary industry, which is consistent with the status of Guangdong Province as a manufacturing province. At the same time, relative to the secondary industry, the development of tertiary industry in Guangdong Province is sensitive to changes in foreign trade, which may be related to the rapid transition of Guangdong Province from the industrial economy stage to the service economy stage, making the coefficient of elasticity of tertiary industry and foreign trade higher. The number of workers employed in this industrial sector has grown yearly along with the expansion of industry in East Java Province. This, combined with the indirect growth of industry, can also influence the provision of employment and maximize employment, helping to combat the high unemployment rate and lessen poverty in the area. The development and performance of the industrial sector are very rapid; this is seen from the output produced from the industrial activity process, which is the result of the value of production. Components of production value are goods produced from the production process^23^.

Elahinia et al.^24^ Conclude that there is a strong causal relationship between manufacturing growth and GDP growth in any nation and analyze the correlation between the manufacturing value added (MVA) and GDP for 92 countries in the period of 1950–1970, 1970–1990 and 1990–2005 using random effects, fixed effects and Hausman tests. According to these empirical results, the relationship between manufacturing output and economic growth is significantly positive.

According to^22^ Study on the Determinants of Manufactured Products Export Performance in Ethiopia using fixed effect, tobit and probit models, the manufacturing industry in Ethiopia is at its lowest stage of development. However, it has registered a 9.7% annual growth rate between the periods 2000 and 2015 on average. This has indicated a significant improvement from the period between1990 and 2000 where the growth was nearly zero. The sector contributes about 5% of the GDP and remains stagnant over time and the export share of the manufacturing sector has remained stagnant at around 15% of total merchandise exports and even exhibited a declining trend despite the growth of the sector. This is one of the indicators of the poor performance of the manufacturing sector in the export market.

Different study has shown the MVA share's relationship to GDP. Various studies using panel data found that credit availability had a significant direct impact on Manufacturing value Added in Kenya, Africa, South Asia and Nigeria^39,40^ and^11^.

Samouel^39^ studied the determinants of industrialization in 35 African countries. Using time series data ranging from 1970 to 2012 and a dynamic panel model, their study revealed that human capital, labor market conditions, the real effective exchange rate, and GDP per capita were found to be the determinants of industrialization in Africa. Moreover, the study depicted that the determinants of industrialization vary between regions on the continent and evolve over time.

The study by^41^ examines the determinants of manufacturing growth in Nigeria using dynamic ordinary least squares methods of econometric analysis. The result of the study indicates that the main determinants of manufacturing growth are foreign direct investment (FDI), the interest rate, the labor force, inflation, and the exchange rate. According to the study, factors affecting the determinant of manufacturing exports were analyzed using the vector error correction model (VECM), and the result indicates that inflation at lag1 and GDP at lag1 and lag2 negatively and positively affect manufacturing exports, respectively, in the short run.

Levinson^5^ estimated the response of manufacturing capacity utilization in Nigeria to changes in key macroeconomic indicators using annual data over the period 1975–2012. Accordingly, a co-integration between the endogenous and exogenous variables was found and variations in manufacturing capacity utilization in Nigeria are largely driven by their own shocks. The study further shows that the exchange rate, interest rate and terms of trade contribute significantly but negatively to variations in manufacturing capacity utilization. The study also presents evidence of the causal impact of manufacturing capacity utilization on the exchange rate and manufacturing capacity utilization on the interest rate, not vice versa, by using the Granger causality test.

The study by^42^ analyzed the drivers of successful industrialization in developing countries. By considering two different periods (1970–1990 and 1991–2014). The empirical analysis reveals that successful industrialization is driven by a combination of factors, including the country’s initial economic conditions, its factor endowments, and other characteristics such as demographic structure and geography. The study also shows that other variables such as the promotion of investments in capital and education; the management of trade and capital openness; financial sector development; and the promotion of both macroeconomic and institutional stability, which policymakers can control, play a crucial role.

Beyene and Singh^31^ studies on the effectiveness of monetary policy on industrial growth in Ethiopia using an autoregressive distributed lag model, revealed that Ethiopia's industrial problems could be tamed by appropriate management of monetary policies. In doing so, the structural shift observed in Ethiopia and its impacts on economic transformation need a close and systematic look. Moreover, the government’s policies towards the exchange rate, interest rate and domestic inflation need a closer check-up, as the unintended results become a pitfall for Ethiopian industry. They conclude that the export share of firms has a positive effect on their growth rate. Again, it is plausible that this effect is of a permanent nature. Firms that are highly export-oriented should show continuously higher growth rates.

**Figure 1:**
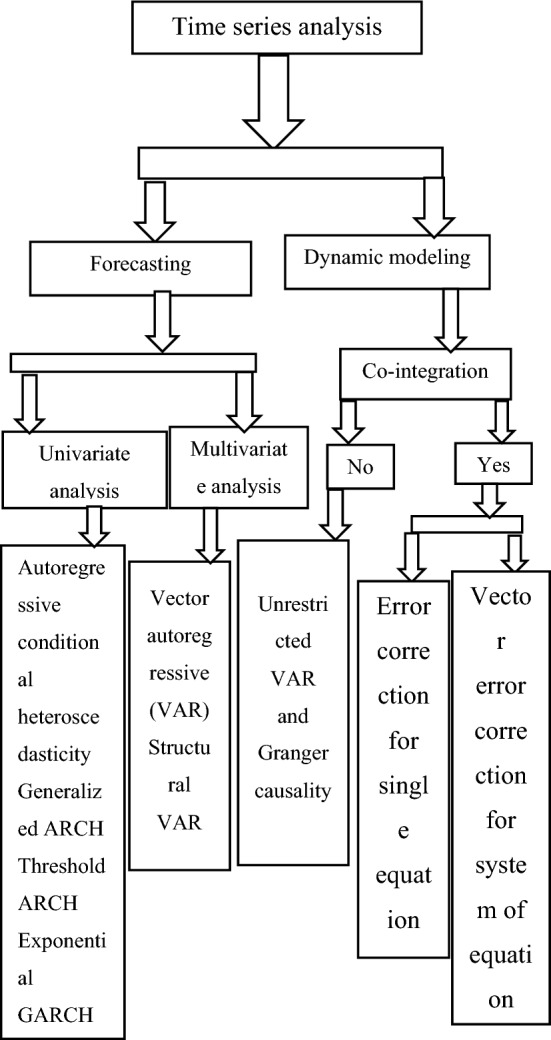
Conceptual frame work for time series analysis.

In the case of this study, annual (yearly) data for the period 1982–2021 were used in the analysis. Multivariate GARCH models are applied in modeling and investigating the volatility and relationship between industry development indicators in Ethiopia. MGARCH is a powerful method to discuss volatility issues in clustered and equally integrated returns series. A kind of general to specific estimation technique is utilized, see the theoretical frame work of time series analysis in Fig. 1.

## Methodology

### Description of study area

The study was conducted in Ethiopia. Ethiopia is a diverse cultural nation and the only country in the horn of Africa that has never been colonized. It is bordered by Sudan to the west, Eritrea to the north, Djibouti and Somalia to the east, and Kenya to the south. The population is concentrated in the northern and southern highlands, with the lowlands in the south-east, south, and west, for the most part, being far more sparsely inhabited.

### Data source and description

The source of data for this study was secondary, and obtained from the World Bank via https://data.worldbank.org/indicator/. World development indicators (WDI) offer metrics for social advancement, lifestyle improvement, economic growth, societal infrastructure development, environmental protection, and governmental performance. WDI is the principal collection of development indicators maintained by the World Bank, compiled from officially sanctioned worldwide sources.

The data existed yearly in the database of the Word Bank. To attain normality, time series analysis needs a large enough number of observations, since 40 observations are enough for multivariate time series modeling^43^. The data was analyzed by Eviews 12 and Ox Metrics 7 statistical software.

### Variables in the study

Industry development (growth) is partially explained by its key indicators, which are manufacturing output (growth), manufacturing imports, manufacturing exports, employment, industry GDP, manufacturing GDP, industrial labor force, wages, and other development indicators. Based on the availability of data and the main target of the study, the variables that would be considered in this study are: industry including construction value added as a proxy of industry growth (INDG), manufacturing value added as a proxy of manufacturing growth (MANG), industry including construction value added (% GDP) as a proxy of industry GDP (INDGDP), manufacturing value added (% of GDP) as a proxy of manufacturing GDP (MANGDP) and medium and high technology (tech) exports as a proxy of manufacturing exports (MANEXP) would be taken as dependent variables; time and the lagged values of dependent variables on the side of independent variables, and also all variables are measured by percent.

### Methods of data analysis

#### Time series analysis

A time series is broadly defined as any series of measurements taken at different times. It can be divided into two major parts: univariate and multivariate time series. Univariate time series analysis uses only the past history of the time series being forecast plus current and past random error terms. Autoregressive integrated moving average (ARIMA) modeling is a specific subset of univariate modeling in which a time series is expressed in terms of past values of itself (the autoregressive component) plus current and lagged values of a "white noise” error term (the moving average component), and the seasonal autoregressive integrated moving average (SARIMA) model is used in non-stationary data^41^. On the other hand, multivariate time series analysis is used when one wants to model and explain the interactions and co-movements among a group of time series variables. This study was concerned with modeling multivariate time series.

Before the models are presented, some basic concepts are mentioned, such as time series plots, a test of stationarity, and transformation if needed.

#### Time series plot

The first step in the analysis of time series is usually to plot the data and obtain simple descriptive measures of the main property of the series via a visual inspection of the time series plot. This may reveal one or more of the following characteristics: seasonality, trends either in the mean level or the variance of the series, long-term cycles, and so on. If any such patterns are present, then these are signs of non-stationarity.

#### Test of stationarity

The foundation of time series analysis is stationarity. A time series ${Y}_{t}$ is said to be strictly stationary if and only if the joint distribution of ${y}_{t1},\cdots ,{y}_{tk}$ is identical to that of ${y}_{t1+t},\dots ,{y}_{tk+t}$ for all $\text{t}$, where $\text{k}$ is an arbitrary positive integer and ${t}_{1},{t}_{2}\dots ,{t}_{k}$ is a collection of $\text{k}$ positive integers. A weaker version of stationarity is often assumed. A time series ${Y}_{t}$ is weakly stationary if both the mean of ${Y}_{t}$ and the covariance between ${Y}_{t}$ and ${Y}_{t-k}$ are time-invariant. More specifically, ${Y}_{t}$ is weakly stationary if $E\left({Y}_{t}\right)=\mu$, which is constant ${\forall }_{t}$, and $\text{Cov}\left({Y}_{t},{Y}_{t-j}\right)={\Gamma }_{j},{\forall }_{t}$ and $\text{j}=\text{0,1},2,.$. In applications, weak stationarity enables one to make inferences concerning future observations^44^.

There are different stationarity tests; among them the following two were used for this study.

##### Augmented Dickey-Fuller (ADF) test

In econometrics, an ADF tests the null hypothesis that a unit root is present in a series^45^. The testing procedure for the ADF test is applied to the model.

1 $$\begin{array}{c}{y}_{t}=\mu +{\phi }_{1}{y}_{t-1}+{\phi }_{2}{y}_{t-2}+\cdots +{\phi }_{p}{y}_{t-p}+{\epsilon }_{t} \\ \Delta {y}_{t}=\mu +{\beta }_{t}+\gamma {y}_{t-1}+{\delta }_{1}\Delta {y}_{t-1}+\cdots +{\delta }_{p-1}\Delta {y}_{t-p+1}+{\epsilon }_{t}\end{array}$$

where $\mu$ is a constant, $\beta$ is the coefficient on a time trend,$\gamma =\left({\sum }_{i=1}^{p} {\phi }_{i}\right)-1,{\delta }_{i}=-{\sum }_{k=i+1}^{p} {\delta }_{k}$ $,\Delta$ is the first difference operator, and $\text{p}$ the lag order of the AR process. Assuming $\mu =\beta =0$, the ADF test is then carried out under ${H}_{0}:\gamma =0$ against ${H}_{1}:\gamma >0$.

The test statistic is: $DFT=\frac{\widehat{\gamma }}{\text{se}(\widehat{\gamma })}$

Where DFT is the ADF test statistic at $\text{t}$ degree of freedom, is compared with the relevant critical value. The ADF statistic is always negative for stationary series. The more negative it is, the stronger the rejections of null hypothesis that there is a unit root at some level of confidence.

##### Phillips Perron (PP) test

The PP test named after^46^ is a unit root test. That is used in time series analysis to test the null hypothesis that a time series is integrated of order 1. It builds on the DFT of the null hypothesis $\rho =1$ in $\Delta {y}_{t}=(\rho -1){y}_{t-1}+{\epsilon }_{t}$. Where $\Delta$ is the first difference operator. Like the ADF test, the PP test addresses the issue that the process generating data for ${y}_{t}$ might have a higher order of auto-correlation than is admitted in the test equation making ${y}_{t}$ endogenous and thus invalidating the Dickey Fuller t-test. Then, PP test makes a non-parametric correction to the t-test statistic.

### Modeling volatility

Volatility provides a measure of the possible variation or movement in particular industry development indicators from period t-1 to t. It indicates how much and how quickly a value changes over time. Volatility connects two principal concepts: variability and uncertainty, with the former describing overall movement and the latter referring to movement that is unpredictable. Wide movements over a short period of time typify the term “high volatility”. The lack of predictability and uncertainty associated with increased volatility may influence both producers and consumers to secure supplies and control input costs. Volatility is often measured as the sample standard deviation.

$$S=\sqrt{\frac{1}{T}\sum_{t=1}^{T} {\left({R}_{t}-\widehat{\mu }\right)}^{2}}$$

where ${R}_{t}$ is the return (i.e. natural logarithm transformed and differenced series) at time t and $\mu$ is the estimated average return over period $T$. Since variance is the square of standard deviation, it makes no difference which ever measure S or ${S}^{2}$ to compare volatilities^47^. Most financial studies involved returns instead of original series.^48^ Gives two main reasons for using returns. First, for average investors, the return of a series is a complete and scale-free summary of the investment opportunity. Second, return series are easier to handle than original series because the former have more attractive statistical properties. Let ${Y}_{t}$ be the series at time index $\text{t}$, which often displays unit-root behavior and thus cannot be modeled as stationary, we often analyze log-returns on ${Y}_{t}$ given as:

2 $${R}_{t}=\text{log}\left(\frac{{y}_{t}}{{y}_{t-1}}\right)=\text{log}\left({y}_{t}\right)-\text{log}\left({y}_{t-1}\right)$$

where ${R}_{t}$ is the $\text{log}$ return series of ${y}_{t}$, and ${y}_{t-1}$ is the series at time $t-1$. The returns for multivariate time series is ${R}_{t}=\left({R}_{1t},\cdots ,{R}_{kt}\right)$ be the log returns of $\text{k}$ series at time $t$ . The squared returns are used as a proxy for volatility.

If there is a large variation from period $t-1$ to $\text{t},{R}_{t}$ be large and we speak of large returns or large volatility. Hence, extreme values for returns reflect extreme variation (volatility). If there is no variation over time,${R}_{t}=\text{log}\left({y}_{t}\right)-\text{log}\left({y}_{t-1}\right)=0$

#### MGARCH models

GARCH models have featured prominently in the analysis of financial time series and are used to study the heteroscedasticity problem. Financial time-series such as foreign exchange rates, inflation rates, and stock prices may exhibit some volatility that varies over time, and this variation is an indicator of ARCH effects or heteroscedasticity problems. Among the numerous specifications of MGARCH models, the most popular seem to be the Vector Error Correction Heteroscedasticity (VECH ), the Baba, Engle, Kraft and Kroner (BEKK) of^49^, Constant Conditional Correlations (CCC) introduced by^50^, and Dynamic Conditional Correlations (DCC) proposed by^51^ and^52^. VECH- and BEKK-GARCH models are the models of conditional covariance matrix, while CCC- and DCC-GARCH are the models of conditional variances and correlations^53^.

Before fitting any GARCH model, it is better to test for the ARCH effect. According to^48^, there are two available methods to test for ARCH effects. The first test is to apply the usual Ljung-Box statistics ${Q}_{(m)}$ to the ${\epsilon }_{t}^{2}$ series. The second test for conditional heteroscedasticity is the Lagrange multiplier test of Engle^54^. In the presence of an ARCH effect or time-varying variance in the residuals of a linear time series model, an ARCH or GARCH model is fitted to the squared residuals of the model in order to remove the heteroscedasticity from the residuals.

The dynamic time dependence (i.e., conditional heteroscedasticity) of ${\varepsilon }_{t}$ is the subject of multivariate volatility modeling. For a k-dimensional time series,${Y}_{t}$ the volatility matrix ${\Sigma }_{t}$ consists of $k$ conditional variances and $\frac{k(k-1)}{2}$ conditional covariance. In other words, ${\Sigma }_{t}$ consists of $\frac{k(k+1)}{2}$ different time-varying elements and the volatility matrix ${\Sigma }_{t}$ must be positive definite for all t.

The complexity of MGARCH models has been a major obstacle to their use in applied work. However, as the dimension of the cross section increases, the number of parameters can become very large in MGARCH models, making estimation increasingly cumbersome. This "dimensionality curse" is general in multivariate time series but is particularly problematic in GARCH models^52^. When the number of parameters becomes too large $(m>9)$ the computation time of the Maximum Likelihood Estimation (MLE) becomes prohibitive, and more importantly, the optimization fails to give a reasonable value.

##### VECH- and BEKK-GARCH models

The difficulty of multivariate volatility model is evident because of the number of parameters involved. For a k-dimensional process, the number of parameters in VECH $(\text{1,1})$ is $\frac{k(k+1)}{2}+$ $2{\left[\frac{k(k+1)}{2}\right]}^{2}$ and the number of parameters in $\text{BEKK}(\text{1,1})$ model is $\frac{k(k+1)}{2}+2[k{]}^{2}$, which is much smaller than the number of parameters for VECH model. Other than the large number of parameters in the model, the other problem with the VECH model is that the conditional covariance matrix may not be positive definite. The BEKK ($p, q, k$) model is given as:

3 $$ \sum_{t} = C^{\prime}C + \sum\limits_{k = 1}^{k} {\sum\limits_{j = 1}^{p} {\Phi^{\prime}_{k} \sum_{t - j} \Phi_{k} } } + \sum\limits_{k = 1}^{k} {\sum\limits_{j = 1}^{q} {\Theta^{\prime}_{k} \varepsilon_{t - j} \varepsilon^{\prime}\Theta_{k} } } $$

where $C$ is a $mxm$ triangular matrix; which is to ensure ${\Sigma }_{t}$ (i.e.Conditional variance covariance matrix) to be definitely positive and is a necessary condition for the estimated variances to be zero or positive, ${\Phi }_{k}$ is kxk matrix which shows the influence of past volatility on the current volatility (i.e. GARCH effect), ${\Theta }_{k}$ is a kxk matrix which shows the effect of past shocks irrespective of their sign (i.e. ARCH effect). $\Phi$ and $\Theta$ are symmetric. The sum,$\sum {\Phi }_{k}+\sum {\Theta }_{k}$ measures the persistence of volatility. Any shock to volatility is permanent if $\sum {\Phi }_{k}+\sum {\Theta }_{k}=1$, that is; past volatility is significant in predicting future volatility. Volatility is explosive if $\sum {\Phi }_{k}+\sum {\Theta }_{k}>1$, meaning, a shock to volatility in one period will lead to even greater volatility in the next period. If $\sum {\Phi }_{k}+\sum {\Theta }_{k}<1$, volatility is neither permanent nor explosive and past volatility prediction is not as such important^55^. The $\text{BEKK}(\text{1,1},\text{k})$ model is:

4 $$ \sum_{t} = C^{\prime}C + \Phi^{\prime}_{k} \Sigma_{t - 1} \Phi_{k} + \Theta^{\prime}_{k} \varepsilon_{t - 1} \varepsilon^{\prime}_{t - 1} \Theta_{k} $$

The model will be stationary if and only if the Eigen values of ${\Phi }_{k}\otimes {\Phi }_{k}+{\Theta }_{k}\otimes {\Theta }_{k}$ are in the unit circle, where $\otimes$ is the kronecker product of two matrices. Partly because of numerical difficulties are so common in the estimation of BEKK models, it is typically assumed that $p=q=1$. In this study the BEKK $(\text{1,1})$ model, its explicit form is given by:

5 $${\Sigma }_{t}=\left(\begin{array}{ccc}{\sigma }_{11,t}& \cdots & {\sigma }_{15,t}\\ \vdots & \ddots & \vdots \\ {\sigma }_{51,t}& \cdots & {\sigma }_{55,t}\end{array}\right)=\left(\begin{array}{ccc}{c}_{11}& \cdots & {c}_{15}\\ \vdots & \ddots & \vdots \\ {c}_{51}& \cdots & {c}_{55}\end{array}\right)+{\left(\begin{array}{ccc}{\phi }_{11}& \cdots & {\phi }_{15}\\ \vdots & \ddots & \vdots \\ {\phi }_{51}& \cdots & {\phi }_{55}\end{array}\right)}{\prime}\left(\begin{array}{ccc}{\sigma }_{11,t-1}& \cdots & {\sigma }_{15,t-1}\\ \vdots & \ddots & \vdots \\ {\sigma }_{51,t-1}& \cdots & {\sigma }_{55,t-1}\end{array}\right) \left(\begin{array}{ccc}{\phi }_{11}& \cdots & {\phi }_{15}\\ \vdots & \ddots & \vdots \\ {\phi }_{51}& \cdots & {\phi }_{55}\end{array}\right)+{\left(\begin{array}{ccc}{\theta }_{11}& \cdots & {\theta }_{152}\\ \vdots & \ddots & \vdots \\ {\theta }_{51}& \cdots & {\theta }_{55}\end{array}\right)}{\prime}\left(\begin{array}{ccc}{\epsilon }_{1,t-1}^{2}& \cdots & {\epsilon }_{5,t-1}{\epsilon }_{1,t-1}\\ \vdots & \ddots & \vdots \\ {\epsilon }_{5,t-1}{\epsilon }_{1,t-1}& \cdots & {\epsilon }_{5,t-1}^{2}\end{array}\right)\left(\begin{array}{ccc}{\theta }_{11}& \cdots & {\theta }_{15}\\ \vdots & \ddots & \vdots \\ {\theta }_{51}& \cdots & {\theta }_{55}\end{array}\right)$$

The interpretation of parameters in the VECH and BEKK models is not easy for the general case with a large p and q, even after imposing several restrictions (i.e., the associated matrices in the model such as ${\phi }_{k}$ and ${\theta }_{k}$ are taken to be diagonal or scalar). The VECH and BEKK models are only applied in low dimensions ($d\le 4$ ). In Diagonal BEKK-GARCH model; the conditional variances are functions of their own lagged values and lagged squared returns, the conditional covariance’s are functions of the lagged covariance and lagged cross-products of the corresponding returns. It reduces the number of parameters estimated by restricting the parameter matrices to be diagonal and addresses the difficulty with VECH by ensuring that the conditional covariance matrix is always positive definite. The BEKK model doesn’t impose the restriction of constant correlation among returns over time. The CCC and DCC-GARCH models are implemented in dimensions larger than 4 in the financial industry^50^.

#### Basic properties of MGARCH family model

Any MGARCH should be unique and stationary; a necessary and sufficient condition to have a unique and stationary solution is ${\sum }_{k=1}^{p} {\phi }_{k}+{\sum }_{k=1}^{q} {\theta }_{k}<1$, Zero in mean: any model in which ${\sigma }_{t}$ is measurable, the mean of ${\varepsilon }_{t}$ is zero. i.e.$E[{\varepsilon }_{t}]=0$, Correlation-free: in GARCH, even though it is conditional, heteroscedasticity is inevitable. Hence, the auto-covariance of any pair of elements from the series is expected to be zero resulting in lack of serial autocorrelation. For $\text{k}>0,{\epsilon }_{t}$ is not correlated with ${\epsilon }_{\text{t}+k}:E\left[{\varepsilon }_{t}{\varepsilon }_{\text{t}+\text{k}}]=0\right.$, and Unconditional variance.

#### Order determination of the MGARCH family model

A model selection criterion considers the "best approximating model" from a set of competing models. An important practical problem is the determination of the GARCH order $p$ and the ARCH order $q$ for a particular series. The Akaike information criterion (AIC) proposed by Akaike (1994), Schwartz Bayesian information criterion (SBIC) proposed by Schwart (1978) and Hannan-Quinn information criterion (HOIC) were employed. The model satisfying minimum AIC or SBIC or HQIC is most representative of the true model. The formal expressions for the above criterion in terms of the log-likelihood are discussed.

$$\text{AIC}=2\text{P}-2\text{ln}(\widehat{L})$$

$$\text{SBIC}=\text{ln}(\text{T})\text{P}-2\text{ln}(\widehat{L})$$

$$\text{HQIC}=\text{lnln}(\text{T})P-2\text{ln}(\widehat{L})$$

where $\hat{L}$ = the maximized value of the likelihood function of the model M, i.e. $\hat{L} = {\text{P}}({\text{R}}/\hat{\theta },{\text{M}})$, where $\hat{\theta }$ are the parameter vlues that maximine the likelibood function; $\text{R}=$ the oberved returns; $T$ = the number of data points in $R$, the number of observations, or equivalently, the sample size; $\text{P}$ = the number of parameters estimated by the model.

#### Parameter estimation of MGARCH models

To be able to predict the volatility of a series, one first has to fit the GARCH model to the time series in question. We can estimate the parameters of MGARCH models by maximum likelihood (ML), assuming that the errors come from a multivariate normal distribution or Student's t-distribution. Both the ML estimator and the quasi-maximum likelihood (QML) estimator, which drops the normality assumption, are assumed to be consistent and normally distributed in large samples. The QML parameter estimates are the same as the ML estimates. As suggested by^48,56^, the estimation of GARCH mode is usually carried out using the MLE method for the VECH and BEKK-GARCH models, while two step estimation method for the CCC and DCC-GARCH models. In MLE, the distributional assumption on residuals is the core point. Financial time series data possess volatility clustering and leptokurtosis characteristics, which lead to the use of different distributional assumptions for residuals such as Gauss and Student-t.

Suppose that ${R}_{\text{t}}(\text{for} t=1,\cdots ,T)$ is the vector of returns with conditional mean, conditional variance matrix and conditional distribution are respectively ${\mu }_{\text{t}}\left({\theta }_{0}\right),{H}_{t}\left({\theta }_{0}\right)$, and $p\left({R}_{t}\mid {\upzeta }_{0} ,{\Omega }_{t-1}\right)$. Where ${\zeta }_{0}=\left({\theta }_{0}{\eta }_{0}\right)$ is r-dimensional parameter vector and ${\eta }_{0}$ is the vector that contains the parameters of the distribution of the innovations ${z}_{t}$ (there may be no such parameter). Importantly, to justify the choice of the estimation procedure, the model to be estimated encompasses the true formulations of ${\mu }_{t}\left({\theta }_{0}\right)$ and ${H}_{\text{t}}\left({\theta }_{0}\right)$.

The most commonly employed distribution in the literature is the multivariate normal, uniquely determined by its first two moments (so that $\zeta =\theta$ since $\eta$ is empty). In this case, the sample log-likelihood is:

6 $${L}_{T}(\theta )=\frac{-1}{2}\sum_{t=1}^{T} \left[k\text{ln}\left(2\pi \right)+\text{ln}\left|{H}_{t}\right|+ {\left({R}_{t}-{\mu }_{t}\right)}{\prime}{H}_{t}^{-1}\left({R}_{t}-{\mu }_{t}\right)\right]$$

where, $\text{T}$ is the number of observations, $\text{k}$ is the number of returns, $\theta$ is the vector of parameters to be estimated.

#### Forecasting volatility

Formally, forecasting volatility could be seen as finding such ${\widehat{\Sigma }}_{t}$ that will minimize the error.

$\varepsilon =f({\Sigma }_{t}-{\widehat{\Sigma }}_{t})$, $\text{where}$ ${\Sigma }_{t}$ is an actual (or observed) volatility over period $t$ and $f(.)$ is an error function. To forecast volatility on a certain time frame one could use data of a smaller time frame and compute the standard deviation. For example, if we are interested in monthly volatility, we can compute the standard deviation of yearly returns. In order to test the forecasting performance of some methods or to compare several methods we should define error functions. The following are the commonly used error functions:

1. Root Mean Square Error (RMSE) = $\sqrt{\frac{1}{N} \sum_{i=1}^{N}({\widehat{\Sigma }}_{t}-{\Sigma }_{t}{)}^{2}}$
2. Mean Absolute Error (MAE) = $\frac{1}{N} \sum_{i=1}^{N}({|\widehat{\Sigma }}_{t}-{\Sigma }_{t}|)$
3. Mean Absolute percent Error (MAPE) = $\frac{1}{N} \sum_{i=1}^{N}\frac{({|\widehat{\Sigma }}_{t}-{\Sigma }_{t}|)}{{\Sigma }_{t}}$
4. Mean Error (ME) = $\frac{1}{N} \sum_{i=1}^{N}({\widehat{\Sigma }}_{t}-{\Sigma }_{t})$
5. Mean Square Error (MSE) = $\frac{1}{N} \sum_{i=1}^{N}({\widehat{\Sigma }}_{t}-{\Sigma }_{t}{)}^{2}$

### Ethics approval and consent to participate

Permission to access the data was obtained from the measure World Bank https://data.worldbank.org/indicator/ via online request. The website and the data used were publicly available with no personal identifier. The proposal was submitted to the Mekdela Amba University ethics committee and they gave permission to conduct the research. All methods were carried out in accordance with relevant guidelines and regulations.

## Results and discussion

### Descriptive statistics

The study is based on secondary data obtained from the World Bank during a 40-year period, collected annually from 1982 to 2021. The data were expressed in natural logarithms to achieve stationarity and make the data have a better goodness of fit for multivariate GARCH models in this study. The data was analyzed by Oxmetrics 7 and Eviews 12 software. In the empirical analysis, INDG, INDGDP, MANG, MANGDP, and MANEXP were used aggregately. Some descriptive statistics, including mean, maximum, minimum, standard deviation, skewness, kurtosis, coefficient of variation (CV), Jarque–Bera statistics, and the corresponding probability values (P-values) of the natural log-transformed and return series under study, are presented in Tables 1 and 2. The return series is the log-transformed and differenced series, which is used as a proxy of volatility.

**Table 1:** Summary results of natural log transformed series.

	lnINDG	lnINDGDP	lnMANG	lnMANGDP	lnMANEXP
Mean	3.30	2.46	3.71	1.55	2.74
Median	3.34	2.40	3.74	1.55	2.91
Maximum	3.79	3.31	4.02	1.99	3.74
Minimum	2.53	1.81	3.22	1.14	1.19
Std.Dev	0.28	0.35	0.17	0.19	0.82
Skewness	-0.70	0.94	-0.61	-0.13	-0.56
Kurtosis	3.71	3.30	3.53	2.65	1.94
CV	0.085	0.142	0.046	0.123	0.299
Jarque–Bera	4.16	6.10	2.97	0.31	3.970
P-values	0.125187	0.047334	0.226116	0.855105	0.137359
Observations	40	40	40	40	40

**Table 2:** Summary results of return series.

	ΔlnINDG	ΔlnINDGDP	ΔlnMANG	ΔlnMANGDP	ΔlnMANEXP
Mean	− 0.002	0.023	− 0.001	0.002	0.049
Median	− 0.005	0.004	0.009	− 0.013	0.012
Maximum	0.367	0.297	0.301	0.337	0.558
Minimum	− 0.495	− 0.246	− 0.391	− 0.362	− 0.381
Std.Dev	0.155	0.119	0.134	0.138	0.202
Skewness	− 0.276	0.209	− 0.747	− 0.174	0.155
Kurtosis	4.936	2.647	4.284	4.026	3.153
CV	− 77.500	5.174	− 134.00	69.000	4.122
Jarque–Bera	6.587	0.486	6.305	1.906	0.195
P-values	0.037128	0.784108	0.042742	0.385674	0.907115
Observations	39	39	39	39	39

Table 1 show that the trend of the series indicated some volatile characteristics during the investigation period. This is highly evident from the fact that the series has a high standard deviation, which is 0.82, 0.35, 0.28, 0.19, and 0.17, respectively. The result showed that the natural log-transformed industry growth at an average rate of 3.30% from 1982 to 2021 in Ethiopia. Moreover, the natural log-transformed average annual manufacturing growth rate was 3.71%, with maximum and minimum values of 4.02 and 3.22, respectively. The mean of the natural log-transformed values for lnINDGDP, lnMANGDP, and lnMANEXP are 2.46, 1.55 and 2.74, respectively. Skewness is negative for lnINDG, lnMANG, lnMANGDP, and lnMANEXP (i.e., the distribution has a longer tail to the left) and positive for lnINDGDP (i.e., the distribution has a longer tail to the right). On the other hand, the kurtosis of series assures that lnINDG, lnINDGDP, and lnMANG are leptokurtic and lnMANGDP and lnMANEXP are platykurtic. The result of Jarque–Bera with corresponding probability shows that all natural log-transformed series except lnINDGDP are normally distributed. Since volatility modeling is applicable for the leptokurtic distribution, the series are returned once.

Table 2 reveals that the return series INDG, INDGDP, MANGDP, MANG, and MANEXP were approximately growing at an average of − 0.002, 0.023, 0.002, − 0.001, and 0.049, respectively, while the standard deviations are almost equal except for MANEXP. Summary statistics show a longer tail to left INDG, MANGDP, and MANG returns and a longer tail to right INDGDP and MANEXP returns, as well as excess kurtosis in all return series except INDGDP, meaning they are highly leptokurtic and INDGDP is platykurtic. The Jarque–Bera test also assures a significant departure from normality due to the excess skewness and kurtosis found in the two returns (INDG and MANG). This implies that the two return series are heteroscedastic.

**Figure 2:**
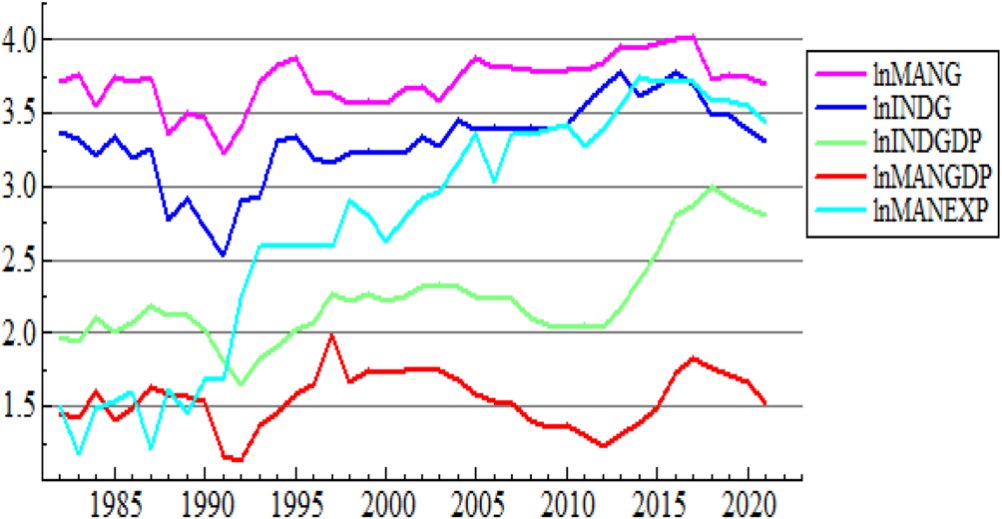
Time plot of the natural Log transformed series.

In most practices, time plots of certain data are very helpful to detect and extract useful insight and pre-information about the data. Thus, inspection should be made on the time plots of the natural log-transformed series. Figure 2 provides the individual plots against the time period covered by the study.

Figure 2 depicts that all natural log-transformed series have some downward trends after 2018, and they showed upward linear trends from 1991 to 2018, but lnMANEXP was stable from 1992 to 1998 and lnINDG had some downward trends from 1982 to 1991. To know which one is increasing more, it is better to see Table 1, the CV row. From the table, the variability of natural log-transformed INDG, INDGDP, MANG, MANGDP, and MANEXP are 8.5%, 14.2%, 4.6%, 12.3% and 29.9%, respectively. Natural log-transformed INDGDP and MANGDP vary almost equally, and MANEXP has the greatest variability above all. Therefore, the variability of INDG (8.5%) is significantly greater than the variability of MANG (4.6%). So industry growth volatility is more than manufacturing growth uncertainty as shown by CV. This might be a motive to see the sort of relationship between major industry development indicators.

From Fig. 2, it can also be seen that there is no clear seasonality. This implies that all the data are non-seasonal and non-stationary. But it should be strongly noticed that only graph inspections are not enough to conclude that the series are non-stationary. Therefore, standard tests for stationarity, which have been discussed previously in methodology, are applied in the analysis as follows:

#### Unit root properties of individual series

In practice, using non-stationary time series is problematic with regard to statistical inference and before making any statistical inference, the stationarity of the series was tested by using ADF and PP tests. The hypothesis to be tested as: H_0_ the series is non-stationary against H_1_ the series is stationary. The results of tests with an intercept at the level and after the first difference are presented in Table 3. The critical values used for the tests are the MacKinnon (1996) critical values.

**Table 3:** Unit root test results.

At level					At first difference			
	Test statistic		Prob*		Test Statistic		Prob*	
Series	ADF	PP	ADF*	PP*	ADF	PP	ADF*	PP*
lnINDG	− 1.76	− 1.83	0.3954	0.3621	− 7.29	− 7.23	0.0001	0.0001
lnINDGDP	− 1.72	− 0.96	0.4116	0.7577	− 4.31	− 4.38	0.0015	0.0013
lnMANG	− 2.51	− 2.51	0.1207	0.1207	− 6.78	− 7.11	0.0001	0.0001
lnMANGDP	− 2.39	− 2.53	0.1514	0.1153	− 6.19	− 6.19	0.0001	0.0001
lnMANEXP	− 1.27	− 1.26	0.6331	0.6405	− 8.22	− 8.14	0.0001	0.0001

Critical Values (5% level): ADF = − 2.8886, PP = − 2.8879.

The test results presented in Table 3 indicate that the null hypothesis (H_O_: the series is unit root) could not be rejected for all series at this level. That is, the respective test statistics are less than critical values at the 5% significance level. In order to determine the order of integration of the non-stationary time series, the same tests were applied to their first differences. Thus, the null hypothesis that the series at first difference contains a unit root could be rejected for all series at the 5% significance level. That is, the absolute value of the respective test statistic is greater than the critical value at the 5% significance level. Therefore, the order of integration is the number of unit roots that are contained in the series so as to be stationary. This implies that all series are integrated in the same order one (I(1)).

### Modeling volatility

To build a volatility model for the return series, the first step is to specify the mean equation based on the information criterion. Once we specify the mean equation, we have to test for ARCH effects using the residuals of the mean equation. If ARCH effects are statistically significant, specifying a volatility model and carrying out a joint estimation of the mean and volatility equations is necessary. The conditional mean specification is, in general, arbitrary for GARCH models of conditional volatility. Various modifications to the conditional means in GARCH models are possible (see, for example,^57^).

#### Checking and testing for the presence of the ARCH effect

**Figure 3:**
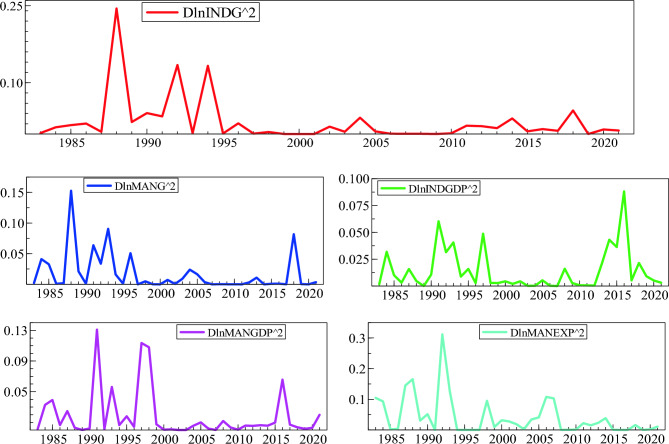
Volatility of industry development indicators.

The squared returns are used as a proxy for volatility and are plotted in Fig. 3. The y-axis limits give an opportunity to compare the volatility of the different series. It seems as though the returns on MANEXP, INDG, MANGDP, MANG and INDGDP are the first, second, third, fourth, and fifth most volatile, respectively. Moreover, the INDGDP seems to be the least volatile. However, the graphical analysis does not give an indication of how volatility reacts to positive and negative news. The first crucial step in the volatility modeling procedure is to assess the presence of ARCH/GARCH effects, which is usually carried out by the Lagrange multiplier (LM) test.

##### ARCH effect test

Based on the residuals from the mean equation, it is possible to test for the existence of the ARCH effect, which will allow continuing the analysis using the GARCH model. In order to estimate the optimal parameters of different GARCH models, it is necessary to set up the presence of ARCH effects. This is done by testing the hypothesis H_O_: there is no ARCH effect (i.e., homoscedasticity) in the residuals Vs H_a_: there is ARCH effect in the residuals using ARCH LM test, and the result is shown in Table 4. The LM-statistic showed the presence of ARCH effects at the 1% and 5% significance levels. Hence, there is volatility clustering, and it is possible to estimate the optimal parameters of MGARCH family models, excluding MANG (i.e., there is no ARCH effect in the return series MANG).

**Table 4:** ARCH Lagrange Multiplier Test of the return Series.

	ΔlnINDG	ΔlnINDGDP	ΔlnMANG	ΔlnMANGDP	ΔlnMANEXP
LM-stat	36.42	36.32	0.29	30.69	4.51
Prob	0.0001*	0.0001*	0.5928	0.0001*	0.0336**

(**)[*]: Statistically significant at (5)^9^ % level.

In the presence of the ARCH effect or time-varying variance in the residuals of a model, an ARCH or GARCH model is fitted to the squared residuals of the model in order to remove the heteroskedasticity from the residuals. Before going to estimate any of the MGARCH models, it is better to determine the best model by using conditional correlation tests and information criteria. But the conditional correlation models (i.e., CCC and DCC-GARCH) did not reach convergence, so these models are not appropriate for this data, and in this case, the researchers fitted only the conditional variance–covariance GARCH model for this study.

##### Lag length selection

If an ARCH effect is found to be significant, the ACFs and PACFs are helpful to determine the order of an ARCH model. This is due to the expectation that is linearly related to it in a manner similar to that of the AR model. In practice, for ARCH modeling a long lag is often needed and this requires estimating a large number of parameters. To reduce the computational burden, a GARCH model with low lags can be helpful. This results in a more parsimonious representation of the conditional variance process. To estimate and evaluate the forecasts of the competing GARCH models, different p and q values for the standard symmetric GARCH models are tested using different statistics such as Akaike Information Criterion (AIC), Bayesian Information Criterion (BIC), and Hannan-Quinn Information Criterion (HQIC) in order to choose the best model based on the in-sample data. Before fitting any MGARCH model, the optimal lag should be determined because too many lags result in loss of degree of freedom, insignificant coefficients, and multi-collinearity, and too few lags result in specification errors (i.e., a poorly specified model). The result is presented in Table 5. The appropriate specification is chosen, and then for these specific p and q, the alternative GARCH models are estimated and tested, and finally one GARCH model is chosen based on forecasting performance.

**Table 5:** Lag Selection for Diagonal VECH- (Part I) and BEKK- (Part II) GARCH Models.

	Lags		T	Log-likelihood	AIC	SBIC	HQIC
Part I	0	1	39	36.89	− 0.661	0.363	− 0.294
	0	2	39	23.23	0.552	2.002	1.073
	1	1	39	56.47	− 1.152*	0.298*	− 0.632*
	1	2	39	51.35	− 0.684	0.936	− 0.103
	2	1	39	58.14	− 1.033	0.588	− 0.451
	2	2	39	− 68.27	5.963	8.010	6.697
Part II	0	0	39	98.31	− 4.324	− 3.727	− 4.110
	0	1	39	104.76	− 4.449	− 3.682	− 4.174
	1	1	39	113.63	− 4.699*	− 3.761*	− 4.363*
	1	2	39	117.29	− 4.682	− 3.573	− 4.284
	2	1	39	113.25	− 4.475	− 3.366	− 4.077
	2	2	39	117.61	− 4.493	− 3.213	− 4.033

*Indicates the lag selected by the criterion.

The results of the information criteria in Table 5 showed that both diagonal VECH (1, 1) and BEKK (1, 1) GARCH models were adopted. But since the values of the information criterion are comparable and small for the diagonal BEKK (DBEKK)-GARCH model, so DBEKK-GARCH (1, 1) is the best multivariate generalized autoregressive conditional heteroscedasticity model adopted for this data. This outcome is a desirable since a smaller number of parameters to estimate remarkably simplify the computational procedure.

#### Estimation of diagonal BEKK-GARCH model

Once the optimal lag length and the appropriate model are selected for the data, the next step is estimating the parameters and obtaining the estimates of each unknown coefficient to interpret the result. This section assesses the ARCH and GARCH effects of each variable’s previous value. ARCH effects measure the impact of previous information on the volatility of the development indicator returns and GARCH effects show the persistence of the return volatility. As shown in Table 6, the sum of ϕ and θ is close to one, which means that the returns are under the influence of shocks. The larger the ARCH coefficient θ is, the greater the impact of the previous shocks. For the GARCH coefficient ϕ, it shows the characteristics of each series itself. Overall, ARCH coefficients were much smaller than GARCH coefficients in this study.

**Table 6:** Estimation of the diagonal BEKK-GARCH model.

	Coefficient	Std.Error	t-statistic	P-values
Mean equation				
ΔlnINDG (${\upmu }_{1}$)	− 0.008	0.016	− 0.487	0.6327
ΔlnINDGDP (${\upmu }_{2}$)	0.026	0.012	2.140	0.0471
ΔlnMANGDP (${\upmu }_{3}$)	0.008	0.011	0.761	0.4572
ΔlnMANEXP (${\upmu }_{4}$)	0.059	0.027	2.181	0.0435
Variance equation				
C_11_	0.065	0.027	2.391	0.0287
C_12_	0.011	0.031	0.359	0.7242
C_13_	0.035	0.034	1.053	0.3069
C_14_	0.014	0.086	0.165	0.8711
C_22_	0.091	0.013	7.194	0.0000
C_23_	0.097	0.020	4.965	0.0001
C_24_	0.004	0.039	0.104	0.9181
C_33_	0.013	0.013	0.986	0.3378
C_34_	0.171	0.246	6.955	0.0000
C_44_	0.000	2.549	0.214	0.83331
Φ_11_	0.622	0.107	5.838	0.0000
Φ_22_	0.598	0.065	9.232	0.0000
Φ_33_	0.583	0.070	8.309	0.0000
Φ_44_	− 0.112	0.202	− 0.555	0.5862
Θ_11_	0.783	0.300	2.606	0.0185
Θ_22_	0.278	0.102	2.716	0.0147
Θ_33_	0.221	0.183	1.208	0.2437
Θ_44_	0.414	0.177	2.335	0.0320

The multivariate diagonal BEKK-GARCH (1, 1) parameter estimations were summarized in Table 6. The estimated model shows that Φ_11,_ Φ_22,_ Φ33_,_ Θ_11,_ Θ_22,_ and Θ_44_ coefficients are significant. The own volatility effect of return series annual industry growth (Θ_11_ = 0.783) is the largest compared with other industry development indicators and the own volatility effect of manufacturing exports (Θ_44_ = 0.414) is the second largest compared with other industry development indicators. And also, the lagged own-volatility effect for industry growth (0.622) and industry GDP (0.598) are the first and second largest compared with others, respectively.

Conditional variance–covariance equations effectively capture the volatility and cross-volatility among the four returns (INDG, INDGDP, MANGDP, and MANEXP) of industry development indicators because most coefficients are statistically significant (Table 6). Specifically, conditional variances and covariance implied by the diagonal BEKK-GARCH (1, 1) specifications are presented in the equations below.

7 $${\Sigma }_{11,\text{t}}=0.004{+0.387\Sigma }_{11 ,\text{t}-1}+0.613{\upvarepsilon }_{1,\text{ t}-1}^{2}$$

8 $${\Sigma }_{12,\text{t}}=0.007{+0.372\Sigma }_{12 ,\text{t}-1}+0.218{\upvarepsilon }_{1,\text{ t}-1}{\upvarepsilon }_{2,\text{ t}-1}$$

9 $${\Sigma }_{13,\text{t}}=0.002{+0.363\Sigma }_{13,\text{t}-1}+0.173{\upvarepsilon }_{1,\text{ t}-1}{\upvarepsilon }_{3,\text{ t}-1}$$

10 $${\Sigma }_{14,\text{t}}=0.001{-0.070\Sigma }_{14 ,\text{t}-1}+0.324{\upvarepsilon }_{1,\text{ t}-1}{\upvarepsilon }_{4,\text{ t}-1}$$

11 $${\Sigma }_{22,\text{t}}={ 0.008+0.358\Sigma }_{22 ,\text{t}-1}+0.077{\upvarepsilon }_{2,\text{ t}-1}^{2}$$

12 $${{\Sigma }_{23,\text{t}}=0.009+0.349\Sigma }_{23 ,\text{t}-1}+0.062{\upvarepsilon }_{2,\text{ t}-1}{\upvarepsilon }_{3,\text{ t}-1}$$

13 $${{\Sigma }_{24,\text{t}}=0.0002-0.067\Sigma }_{24 ,\text{t}-1}+0.115{\upvarepsilon }_{2 ,\text{ t}-1}{\upvarepsilon }_{4,\text{ t}-1}$$

14 $${{\Sigma }_{33,\text{t}}= 0.0108+0.340\Sigma }_{33 ,\text{t}-1}+0.049{\upvarepsilon }_{3,\text{ t}-1}^{2}$$

15 $${\Sigma }_{34,\text{t}}={0.002-0.065\Sigma }_{34 ,\text{t}-1}+0.092{\upvarepsilon }_{3,\text{ t}-1}{\upvarepsilon }_{4,\text{ t}-1}$$

16 $${\Sigma }_{44,\text{t}}={0.030+0.013\Sigma }_{44 ,\text{t}-1}+0.172{\upvarepsilon }_{4,\text{ t}-1}^{2}$$

The equations above are used as a representation of the diagonal BEKK-GARCH model constructed for the period 1982–2021 using four industry development indicators. The model takes into account both its own specific volatility and cross-volatility impacts. The log likelihood for this diagonal BEKK-GARCH model is 113.64; the higher log likelihood for multivariate GARCH models makes them suitable for presenting the interaction among the return volatility. First, we compare the equations of own volatilities. The constant of conditional variance ${\Sigma }_{44,\text{t}}$ (0.03) is larger than any other conditional variance, which suggests greater volatility in manufacturing exports.

From these empirical results, we conclude that there is strong evidence for a higher GARCH effect and the presence of a weaker ARCH effect. Equations show a statistically significant co-variation in shocks, which depends more on their lags than on past errors. Consequently, development indicator shocks are influenced by past information.

Own-volatility effects (ARCH effects) are positive and significant for three return development indicators (INDG, INDGDP, and MANEXP). The effect is higher for INDG (0.613) and MANEXP (0.172) than for INDGDP (0.077) and MANGDP (0.049). These coefficients show the volatility persistence for each indicator in terms of its own past errors.

As for cross-volatility effects, the strongest ARCH effect (0.324) is detected between INDG and MANEXP, which shows that previous information from manufacturing exports affects the growth of industry. The ARCH effect (0.218) is the second strongest, which is between industry growth and industry GDP, and the weakest ARCH effect (0.062) is detected between industry GDP and manufacturing GDP. The cross-volatility effects are higher than the own-volatility effects in INDGDP, MANGDP, and MANEXP. However, past volatility shocks in INDG have less effect on cross-volatility than its own volatility shock. These results suggest that MANGDP is the least vulnerable indicator to outside shocks.

The lagged own-volatility persistence effects (GARCH effects) are INDG (0.387), INDGDP (0.358), MANGDP (0.340), and MANEXP (0.013). These results suggest that INDG derives more of their volatility and persistence from within themselves. Moreover, the own volatility effects for four indicators do not remain within a tight range. This further implies that each indicator of growth faces a different risk-return profile and different levels of vulnerability to outside conditions.

For the return industry growth (INDG), the lagged cross-volatility persistence goes from 0.372 (INDGDP) to -0.070 (MANEXP), and in INDGDP, it goes from 0.372 (INDG) to -0.067 (MANEXP). Conversely, in MANGDP, the cross-volatility persistence varies between 0.363 (INDG) and -0.065 (MANEXP), while in MANEXP it goes from -0.070 (INDG) to 0.065 (MANGDP). Hence, in terms of cross-volatility persistence, the least influential indicator in the study is MANEXP, while the most influential would appear to be INDGDP. On the other hand, past volatility shocks in INDGDP have the greatest effect on the future volatility of INDG.

It is an important finding here that although cross-volatility persistence is heterogeneous for four indicators. The influence of lagged covariance on future covariance is positive for all pairs but negative for MANEXP pairs. The analysis implied that the magnitude of the cross-volatility persistence of the development indicators.

The plots for the conditional variance covariance estimated by the diagonal BEKK model are illustrated below. They suggest that the co-movements of the industry development indicators in Ethiopia display an extremely volatile trend for the study period.

**Figure 4:**
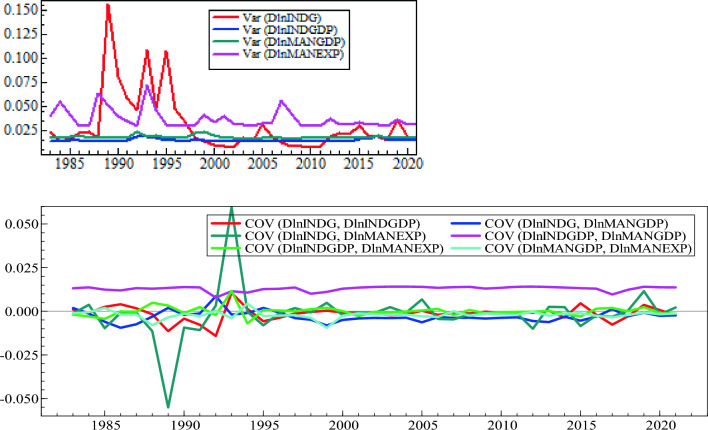
Conditional Variance and Covariance’s plot for Industry development indicators.

**Figure 5:**
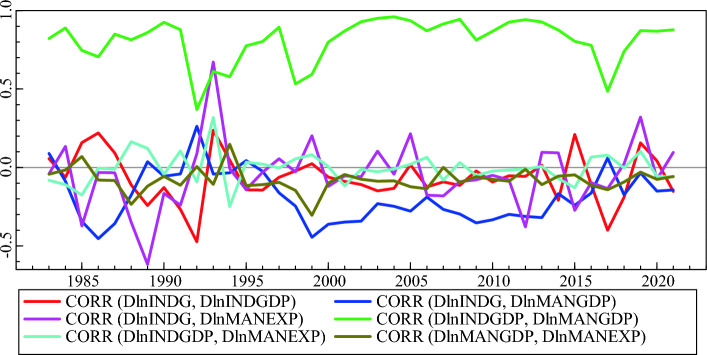
Conditional correlation plot for Industry development indicators.

The conditional variance, covariance, and correlation of four industry development indicators are shown in Figs. 4 and 5. The plot indicates that there is a positive association between INDGDP and MANGDP, but all the other relationships between the return series vary between positive and negative values. Taking manufacturing GDP and manufacturing exports into account, as the share of manufacturing GDP increases, the general level of industry GDP also increases. The positive relationship between industry GDP and manufacturing exports means that an increase in the share of manufacturing exports leads to an increase in industry gross domestic product in a modernized system, which affects the health of one’s country's economy in a positive way, especially in Ethiopia, which depends heavily on import goods.

#### Diagonal BEKK-GARCH model adequacy checking

Checking the adequacy of MGARCH models is essential in identifying whether a well-specified MGARCH model can obtain reliable estimates and inferences. Starting by looking at the residuals, they should be following a white noise process. The ACF plot for the squared standardized residuals, given in Fig. A.1 (Appendix), shows that the residuals are not auto-correlated for all return series. By looking at the ACF graphs, it can be determined whether the processed data has any trace of correlation in the residuals. If more than 5% of the lags in the autocorrelation plot fall outside the confidence interval, the residuals cannot be treated as white noise. In this model, almost all lags out of 20 fall within the confidence level. It can therefore be concluded that the residuals do not suffer from heteroscedasticity. The portmanteau test of white noise in Table 7 confirms the graphical view. After a diagonal BEKK-GARCH model had been fitted, the Ljung Box statistics (Q-test) and Li Mcloed (Q*-test) were employed for residuals to check for the model adequacy in Table 7 to test the null hypothesis of no serial correlation. The overall result showed that the model performed well statistically since p-values were insignificant. It means that the null hypothesis, which says there is no serial correlation in the squared residual, was accepted.

**Table 7:** Multivariate Portmanteau Test for DBEKK-GARCH.

Lags	Q-stat	P-values	Adj Q-stat	P-values
1	18.11	0.2018	18.06	0.2043
2	32.09	0.3633	32.14	0.3611
3	53.34	0.2128	52.99	0.2226
4	83.18	0.0376	81.41	0.0501

*The test is valid only for lags larger than the system lag order.

##### The ARCH LM test of residuals

The results from the ARCH LM test of the return series in Table 4 indicate the presence of volatility clustering in the data. The next model diagnostic is to determine whether there are still ARCH effects left in the residuals or not. If there are still unexplained effects left, this means that the chosen model suits the sample poorly since the return series suffers from heteroscedasticity according to the first ARCH LM test. Table A.1 in the appendix, Lagrange multiplier test approach, shows insignificant p-values for all standardized residuals. The conclusion of the LM tests is that a model captures volatility clustering successfully.

##### Normality of standardized residuals

GARCH estimates could be sensitive to the assumption of normally distributed errors. So it is important to check the extent to which this assumption is correct. Since the standardized errors are not directly observable and have to be estimated, chi-squared based tests such as the skewness and kurtosis tests for normality are not strictly applicable. Instead, it is advisable to plot the quantiles of the standardized residuals against the quantiles of the standardized normal distribution (Q-Q plot) and check if they are similar^58^.

**Figure 6:**
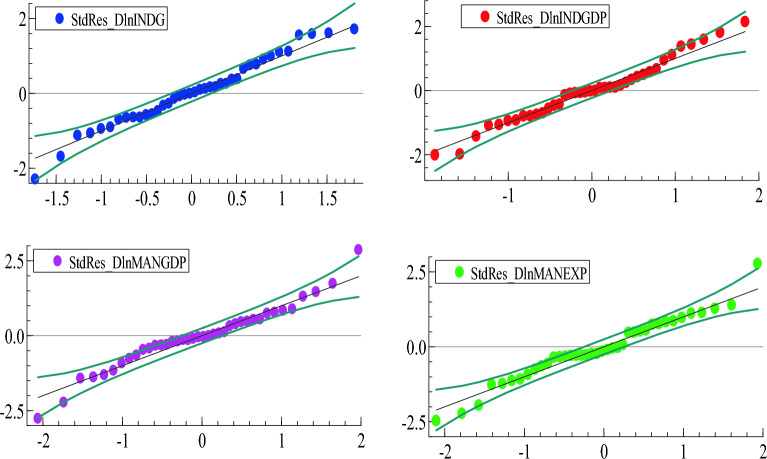
Normality (Q-Q) plot of standardized residuals, diagonal BEKK-GARCH model.

Figure 6 shows that in all return series, the points form a roughly straight line, with approximately half the data below 0 and the other above 0. Therefore, standardized errors are approximately normally distributed, except for deviations at the beginning and end of the tails. The two outlier points, as shown in the residual Q-Q plots of the return series of manufacturing GDP and manufacturing exports do not have an impact on the model because the predicted response, estimated coefficient, and hypothesis test result are not affected by the inclusion of the point. Therefore, the data point is neither influential nor an outlier, but it does have leverage. This is confirmed in Fig. A.2 (Appendix), a histogram of the standardized residuals from the diagonal BEKK-GARCH (1, 1) model.

#### Forecasting from the DBEKK-GARCH model

##### Out of samples, volatility forecasting

Forecasting is a deep-seated ambition of time series analysis or developing a time series model. The previous discussions validated that the diagonal BEKK-GARCH (1, 1) model is the paramount model to suitably describe the volatility of the return series of this study. This section conducts an examination of the forecasting accuracy of the fitted model and then makes a forecast for 2022–2026. A five year forecast is made for conditional variances, and the results are plotted in Fig. 6.

**Figure 7:**
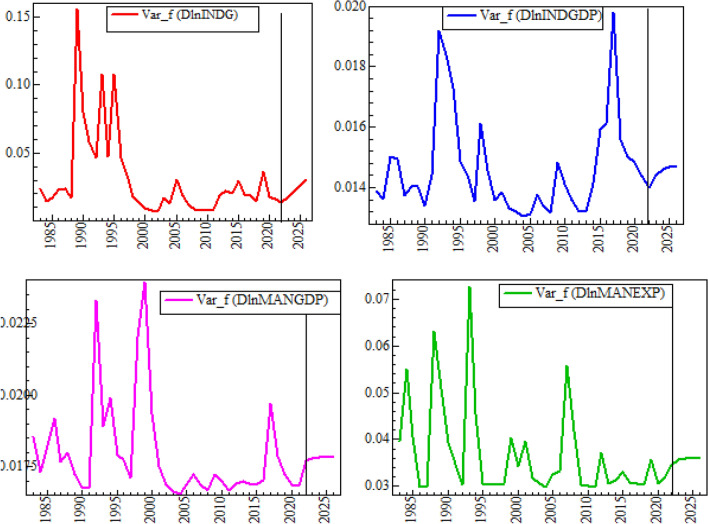
In (1982 to 2021) and Out (2022 to 2026) of Samples Forecasted Volatility.

Figure 7 exhibits the time series forecast of volatility in INDG, INDGDP, MANGDP, and MANEXP for the next five years. The forecast indicates that there is likely to be stability in all variables for the next five years. This implies that the returned series are expected to increase at a low rate, but the return series INDG is increasing very fast. Therefore, analyzing and forecasting volatility is helpful as it informs investors of the measures of risk involved in holding an asset because investors are not only interested in the average returns.

## Conclusions

The general objective of the study was to assess the volatility and relationship between industry development indicators in Ethiopia using a multivariate GARCH model based on World Bank data from 1982 to 2021, and the data were transformed to natural logarithms (by adding a constant for the variables that have a negative value) for easy analysis. However, without understanding the direction of relationships between industry development indicators, it is not possible to draw important lessons for policy-making purposes in an effort to pursue more effective policies that promote industry growth. Initially, all components were identified as non-stationary using Augmented Dickey-Fuller (ADF) and Phillips-Perron (PP) unit root tests. Thus, the result showed that all the series were, firstly non-seasonal, some are upward trended, volatile and non-stationary. The problems of non-stationarity were solved by differencing all the series once.

The ARCH Lagrange Multiplier test showed evidence of volatility clustering, and therefore the DBEKK-GARCH model was estimated excluding annual percent of manufacturing growth because there is no ARCH effect in manufacturing growth.

Among the four multivariate GARCH models, DBEKK-GARCH (1, 1) is found to be the appropriate volatility model, which is adopted by using different tests and information criteria.

The model takes into account both its own specific volatility and cross-volatility impacts. And manufacturing GDP is positively correlated with industry GDP and manufactured exports, and industry growth is positively correlated with manufactured exports.

Since ARCH coefficients are lower than GARCH term coefficients, there is evidence of a high rate of change in conditional volatility and significant time dependence.

Industry growth is the largest volatility effect in both cases (i.e., ARCH effect and the lagged own volatility (GARCH effect)) compared with others, as indicated by the diagonal BEKK-GARCH (1, 1) model.

Finally, the forecast showed an increment in industry growth, industry GDP, and manufacturing exports, while stability was showed in the volatility of manufacturing GDP.

## Recommendations

- As a result of these findings, policy-makers should prioritize the industry sector's development by enacting the following incentive conditions: The government and investors must work together to change and stabilize the volatile manufacturing exports and industry growth by stabilizing recurring policy changes and other unstable conditions. Policy makers should focus as much on industry and manufacturing growth as possible and there must be a national consensus to reduce imports of manufacturing goods into the country by improving production and increasing manufacturing exports.
- We recommend and encourage future researchers studying the forecasting performance of MGARCH models to pay particular attention to the measurement of realized volatility and employ high-frequency data whenever feasible.

## Limitation of the study

This study is limited to some extent due to inexplicitly recorded data and small number of observations on some variables like manufacturing imports, employment, wages, industrial labor force, skills, and education.

## Significance of the study

Fitting and investigating the relationship and volatilities between industry development indicators are necessary for several reasons. First, to overcome the debates existing in their relationship and the uncertainty about future growth, which causes unexpected gains and losses in trade and industry, thus discouraging long-term contracts and investment, and to serve as a mirror in showing the trends and the major forces causing manufacturing industry growth, which helps policy-makers with proper ways of intervention for working the growth of the manufacturing industry and the whole industry growth.

### Supplementary Information

Supplementary Information.

## Data Availability

The datasets analyzed during the current study are available in the World Bank repository, https://data.worldbank.org/indicator/.
