# Supplementary material for: Modeling and forecasting the volatility of some industry development indicators in Ethiopia using multivariate GARCH models
Source: Sci Rep. 2024 Jun 22;14:14395. doi: 10.1038/s41598-024-64749-3 (PMC11193759; doi:10.1038/s41598-024-64749-3)
Supplement: Supplementary file 1 — Supplementary Information. [file 41598_2024_64749_MOESM1_ESM.docx]

# **APPENDIX**

Table A.1: ARCH-LM test for residuals of DBEKK-GARCH model

|  | ResDlnINDG | ResDlnINDGDP | ResDlnMANGDP | ResDlnMANEXP |
| --- | --- | --- | --- | --- |
| LM-stat | 0.930 | 0.004 | 0.023 | 0.645 |
| Prob. | 0.3361 | 0.9517 | 0.8804 | 0.4218 |

Table A.2: summary of data

|  | Variables | | | | |
| --- | --- | --- | --- | --- | --- |
| year | Industry growth | Industry GDP | Manufacturing growth | Manufacturing GDP | Manufactured exports |
| 1982 | 28.91868 | 8.833303 | 41.10125 | 4.269014 | 4.529173 |
| 1983 | 27.65743 | 8.483487 | 42.94617 | 4.129561 | 3.275708 |
| 1984 | 24.66514 | 10.14246 | 35.05841 | 4.9491 | 4.445149 |
| 1985 | 28.14104 | 9.174057 | 42.00723 | 4.059937 | 4.656819 |
| 1986 | 24.39345 | 9.704191 | 40.74369 | 4.397055 | 4.946342 |
| 1987 | 26.05642 | 11.00483 | 42.54385 | 5.146067 | 3.379398 |
| 1988 | 15.88937 | 10.27592 | 28.78366 | 4.871669 | 5.077071 |
| 1989 | 18.5186 | 10.31786 | 33.29391 | 4.833419 | 4.262595 |
| 1990 | 15.13104 | 9.303867 | 32.28312 | 4.62518 | 5.347526 |
| 1991 | 12.56872 | 7.277105 | 25.07945 | 3.221012 | 5.347526 |
| 1992 | 18.13786 | 6.094016 | 30.1334 | 3.113509 | 9.347526 |
| 1993 | 18.89781 | 7.454667 | 40.72233 | 3.946226 | 13.34753 |
| 1994 | 27.20945 | 8.183458 | 46.24278 | 4.273618 | 13.34753 |
| 1995 | 28.12629 | 9.286202 | 47.88538 | 4.886132 | 13.34753 |
| 1996 | 24.34159 | 9.783691 | 38.22588 | 5.212365 | 13.34753 |
| 1997 | 23.67811 | 12.20217 | 37.96525 | 7.301088 | 13.30065 |
| 1998 | 25.20639 | 11.57038 | 35.38387 | 5.256874 | 18.10878 |
| 1999 | 25.48374 | 12.19479 | 35.70586 | 5.733774 | 16.41055 |
| 2000 | 25.34871 | 11.41047 | 35.657 | 5.605245 | 13.73129 |
| 2001 | 25.11418 | 11.92793 | 38.80758 | 5.776228 | 16.22728 |
| 2002 | 28.32587 | 12.74028 | 39.80924 | 5.766491 | 18.61401 |
| 2003 | 26.47558 | 12.8995 | 36.20607 | 5.770038 | 19.51068 |
| 2004 | 31.64521 | 12.68554 | 42.26536 | 5.350706 | 23.48301 |
| 2005 | 29.43117 | 11.78883 | 48.03492 | 4.842961 | 28.75332 |
| 2006 | 30.16407 | 11.59232 | 45.30053 | 4.637738 | 20.69659 |
| 2007 | 29.52326 | 11.5895 | 44.93025 | 4.579682 | 28.52703 |
| 2008 | 30.13033 | 10.20685 | 44.26184 | 4.108135 | 28.85756 |
| 2009 | 29.67331 | 9.68348 | 43.62498 | 3.880256 | 29.49909 |
| 2010 | 30.81716 | 9.435128 | 44.20769 | 3.973906 | 30.48217 |
| 2011 | 35.01086 | 9.664642 | 44.23839 | 3.682551 | 26.25499 |
| 2012 | 39.63782 | 9.476138 | 46.80447 | 3.420061 | 29.69926 |
| 2013 | 44.10272 | 10.94456 | 51.93358 | 3.702189 | 34.58343 |
| 2014 | 37.04233 | 13.47132 | 51.63703 | 3.991325 | 42.07409 |
| 2015 | 39.84619 | 16.29779 | 53.22264 | 4.404619 | 41.2687 |
| 2016 | 43.94405 | 21.93257 | 54.90063 | 5.691399 | 40.76614 |
| 2017 | 40.57214 | 23.58191 | 55.65523 | 6.186589 | 41.12975 |
| 2018 | 32.73995 | 27.3057 | 41.82134 | 5.827617 | 36.22812 |
| 2019 | 32.55572 | 24.82237 | 42.69717 | 5.594369 | 36.22812 |
| 2020 | 29.63511 | 23.10229 | 42.51238 | 5.303281 | 34.59571 |
| 2021 | 27.25807 | 21.85234 | 40.09975 | 4.605303 | 31.12832 |


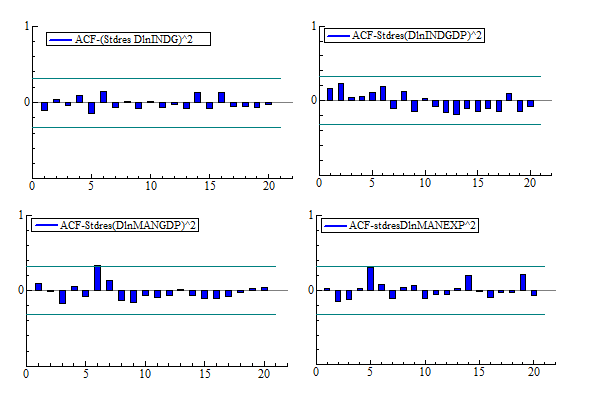


Figure A. 1: ACF of squared standardized residuals of the return series from Diagonal BEKK model

Figure A. 2: Histogram of the standardized residuals from Diagonal BEKK model
